# Oestrogenic Endocrine Disruptors in the Placenta and the Fetus

**DOI:** 10.3390/ijms21041519

**Published:** 2020-02-23

**Authors:** Zi-Run Tang, Xue-Ling Xu, Shou-Long Deng, Zheng-Xing Lian, Kun Yu

**Affiliations:** 1 Beijing Key Laboratory for Animal Genetic Improvement, National Engineering Laboratory for Animal Breeding, Key Laboratory of Animal Genetics and Breeding of the Ministry of Agriculture, College of Animal Science and Technology, China Agricultural University, Beijing 100193, China; S20183040519@cau.edu.cn (Z.-R.T.); SY20183040655@cau.edu.cn (X.-L.X.); lianzhx@cau.edu.cn (Z.-X.L.); 2 CAS Key Laboratory of Genome Sciences and Information, Beijing Institute of Genomics, Chinese Academy of Sciences, Beijing 100101, China; popo84350746@163.com; 3 Institute of Laboratory Animal Sciences, Chinese Academy of Medical Sciences, Ministry of Health, Beijing 100021, China

**Keywords:** placenta, fetus, estrogenic endocrine disruptors

## Abstract

Endocrine disrupting chemicals (EDCs) are exogenous substances that interfere with the stability and regulation of the endocrine system of the body or its offspring. These substances are generally stable in chemical properties, not easy to be biodegraded, and can be enriched in organisms. In the past half century, EDCs have gradually entered the food chain, and these substances have been frequently found in maternal blood. Perinatal maternal hormone levels are unstable and vulnerable to EDCs. Some EDCs can affect embryonic development through the blood-fetal barrier and cause damage to the neuroendocrine system, liver function, and genital development. Some also effect cross-generational inheritance through epigenetic mechanisms. This article mainly elaborates the mechanism and detection methods of estrogenic endocrine disruptors, such as bisphenol A (BPA), organochlorine pesticides (OCPs), diethylstilbestrol (DES) and phthalates (PAEs), and their effects on placenta and fetal health in order to raise concerns about the proper use of products containing EDCs during pregnancy and provide a reference for human health.

## 1. Introduction

An endocrine-disrupting compound is defined by the U.S. Environmental Protection Agency (EPA) as “an exogenous substance responsible for homeostasis, reproduction, and development that interferes with synthesis, secretion, transportation, metabolism, binding or elimination of natural blood-borne hormones in the body” [1]. In brief, an endocrine-disrupting substance is a compound that alters the hormonal and homeostatic systems. Molecules identified as endocrine disruptors are highly heterogeneous [2]. The sources of endocrine disrupting chemicals exposure are usually diverse and widely distributed in our daily life. EDCs are mostly man-made, including organochlorine pesticides (OCPs), plastics (e.g., bisphenol A (BPA)), cosmetic products, plasticizers (e.g., phthalates (PAEs)), and pharmaceutical agents (e.g., diethylstilbestrol (DES)) [3]. Natural chemicals found in the human diet (e.g., phytoestrogens, including genistein, daidzein, and coumestrol) also act as EDCs [2]. Since the late 1990s, the European Union (EU) has been working on policies related to EDCs that aim at phasing out endocrine disruptors in industrial chemicals, plant protection products, and biocides [4]. In 2017, the European Commission announced that it would start regulating consumer products, such as toys, cosmetics, and food packaging to minimize the exposure of EU citizens to endocrine disruptors [5]. EDCs may have adverse effects: they can interfere with significant life processes, such as sexual development, growth, and propagation, as well as the development of a living fetus [6].

The placenta is an important barrier for fetal protection during pregnancy. The placenta can produce human chorionic gonadotropin (hCG) and also participate in the exchange of gases, nutrients, and waste materials between mother and fetus. The human placenta develops primarily from trophoblast ectoderm cells outside the blastocyst. Cytotrophopblasts can be divided into villous cytotrophoblasts and extravillous trophoblasts (EVTs). Villous trophoblast cells continually fuse to form the human syncytiotrophoblasts [7]. EVTs invade the endometrium in the upper third of the myometrium, anchoring the fetus to the uterine wall. In addition, EVTs can invade the uterine spiral arteries (SAs), resulting in the remodeling of SAs, which provide the uterus with nutrient-rich blood [8]. Notably, the fetus may be indirectly affected by various EDCs through the placenta [9]. During this period, hormone and protein levels will change and balance as cells begin to grow and differentiate. When EDCs are transferred into the fetus through the placenta, the equilibrium state of the hormonal environment will be broken, due to the sensitivity of the immature fetus to EDCs, resulting in miscarriage, fetal growth retardation, and preeclampsia [10,11,12].

**Figure 1:**
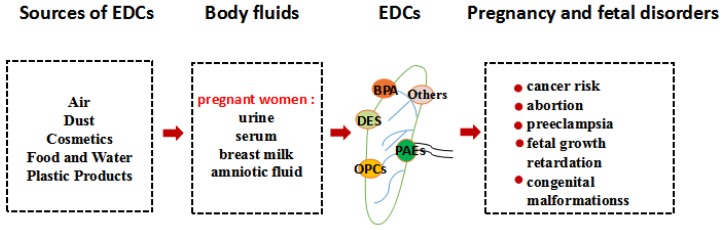
The transfer routes of several endocrine disrupting chemicals (EDCs) and the damages to pregnant women and fetuses. Pregnant women are exposed to EDCs from the air, food, and products which damage the placenta through body fluids, thereby causing health problems.

As shown in Figure 1, EDCs can enter pregnant women in different ways, e.g., through the food chain, the skin, or the respiratory tract. The mother may drink water or eat foods contaminated with EDCs. For example, phthalates are connected to polymer molecules through van der Waals forces and oxygen bonds in plastics products, which maintain relatively independent chemical properties of each other. With time, phthalates can slowly leak out of plastic products, into water, or food packaged in plastic [13]. Concentrations of BPA indoors have been estimated to be 1.46 mg/kg [14]. Mothers sometimes breathe dust in rooms with wallpaper or flooring containing EDCs or touch products made with BPA. A number of studies have revealed that some EDCs can be detected in the serum, amniotic fluid, breast milk, and urine of pregnant women [15,16]. Some of these may accumulate in placental tissues, and impair placental function, including plasticizers, organochlorine pesticides, personal care products, or naturally occurring EDCs found in plants. BPA exposure can significantly alter the methylation of imprinted genes in the mouse placenta, causing placental abnormality [17]. Clearly, the placenta is essential for the normal development and growth of the fetus. Subtle changes in placental function during the first trimester of pregnancy can change the fetal developmental trajectory, which affects various fetal physiological systems, such as the nervous system and immune system. Under this possible influence, the fetus is prone to disease, leading to congenital malformations [18]. Furthermore, chemical exposures to the mother may reach the fetus as a result of circulating blood through the placenta. Plasticizers such as phthalates and BPA have been reported to reach the fetus by blood circulating through the placenta because of the embryo’s lack of adequate defense mechanisms [19]. Non-persistent organic pollutants (non-POPs) were also detected in meconium samples, meaning that there is a limit to the ability of the placenta to act as a barrier to these chemicals and indicating that they had already reached the developing fetus and been absorbed [20]. Even relatively modest concentrations, some lipid-soluble chemicals, such as organochlorine pesticides, are able to pass the placental barrier, through a simple mechanism of passive diffusion. One study showed that after crossing the placental barrier, organochlorine pesticides can also pass through the blood-brain barrier of a still immature fetus [21]. Fetal exposure to EDCs has been associated with physical developmental, and eventually may adversely compromise their reproductive and hormonal systems, as well as cause cancer [22]. Although several adverse effects of EDCs have been reported in the reproductive process, most are focused on child growth and maternal health. The damages on the health of the placenta and fetus are unclear. Here, we review several major estrogen endocrine disruptors in the body fluids of pregnant women, then dissect the influences of placenta and fetal health in order to effectively prevent diseases caused by EDCs in mothers and fetus.

## 2. Detection Methods

Pregnant women are a unique population who are exposed to endocrine disruptors during pregnancy that can affect the baby through body fluids. Liquids such as urine, maternal milk, serum, and amniotic fluid are currently the most commonly used substances to assess the EDCs in the body [16,23]. Its widespread use has led to the detection of BPA in low doses in pregnant women. The measurement of organic pollutants (POPs) in mothers is usually via breast milk or serum samples [24]. Since the phthalates are rapidly metabolized into monoester metabolites in the body and then excreted in the urine, monitoring urinary phthalate metabolites is a commonly used method to investigate pregnant women’s exposure to phthalates [25,26]. Phytoestrogens, such as daidzein and genistein, have been found in the serum and amniotic fluid of pregnant women [27]. Experiments have shown that genistein could be transplanted in human and rat placentas [28,29]. Scientists are continuously improving analytical methodologies in order to be able to identify environmental endocrine disruptors in pregnant women [30]. Among them, mass spectrometry (MS) has the characteristics of high sensitivity and strong qualitative ability. However, for some compounds, such as alkylphenols, synthetic sterols, and bisphenol A, mass spectrometry requires sample preparation and cleaning and derivatization steps, which makes this method inefficient for detecting analyses. Furthermore, gas chromatography (GC) has a highly effective separation effect for each component in the mixture. With the development of science and technology, the GC combines this with MS to increase the reliability in the detection of environmental endocrine disruptors. Therefore, gas chromatography-mass spectrometry (GC-MS) technology has been greatly developed. In addition, liquid chromatography-mass spectrometry (LC-MS) has also begun to be applied. High performance liquid chromatography (HPLC) has a wide range of applications and high sensitivity, and the processed samples are not damaged and easy to recover. However, HPLC is not as sensitive as gas chromatography. Table 1 lists the detection methods of some EDCs in the body fluids of pregnant women.

## 3. Endocrine Disrupting Chemicals

### 3.1. Bisphenol A

BPA is an industrial chemical that has been used to make certain plastics and epoxy resins. More specifically, food packaging bottles are mostly made of polycarbonate plastics, while epoxy resins are extensively used in composite materials, such as beverage cans, bottle tops, and water supply lines. In addition, BPA is also found in other daily items, such as thermal printing papers, medical equipment, coating powders, sunglasses, baby bottles, and sippy cups [42]. It is a colorless solid, poorly soluble in water, but soluble in organic solvents, such as ether, acetone, and alcohol. In our life, we are exposed to BPA toxicity directly through foods packaged with BPA products. Children and adults who eat canned foods were found to have higher urinary BPA levels in the past 24 h [43]. On the other hand, we are indirectly exposed to BPA contamination through the environment [44].

**Figure 2:**
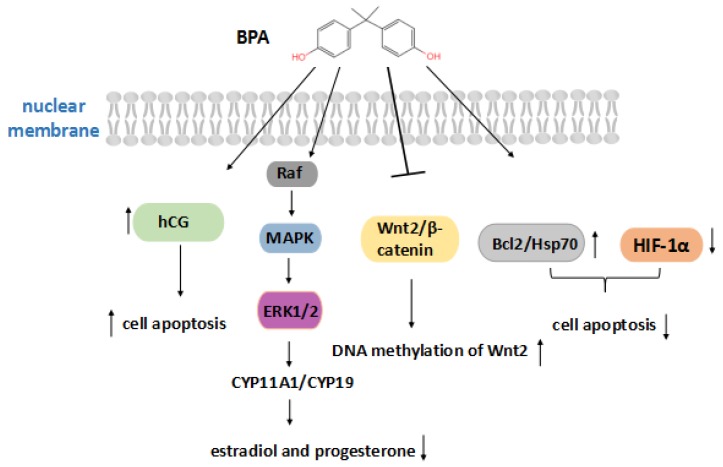
BPA action on placental growth. The arrow facing up indicates an increase, and the arrow facing down indicates a decrease, due to BPA. Bisphenol A increases the secretion of hCG and cell apoptosis, or activates the ERK signaling pathway to reduce estradiol and progesterone hormones, and also inhibits the expression of Wnt2/β-catenin. On the other hand, BPA can increase the levels of Bcl-2 and Hsp70 and reduce the level of HIF-1α to reduce apoptosis.

In fact, exposure to BPA during early embryonic development can lead to numerous development abnormalities. According to Figure 2, interfering with placental production of hCG may be a means by which BPA affects the fetus. The roles of hCG during pregnancy include the promotion of the invasion and differentiation of trophoblast cells, the growth of the placenta, and angiogenesis in the uterine vasculature. BPA can directly act on the placenta, leading to abnormal labyrinthine development of trophoblast cells, which can severely affect the hCG secretion of the early trimester trophoblast and increase cell apoptosis [45]. However, some experiments have shown that BPA increased the levels of anti-apoptotic proteins like Bcl-2 and Hsp70, and decreased HIF-1α levels in BeWo cells, reducing apoptosis [46]. Furthermore, a low dose of BPA (1–1000 nM) could activate the ERK signaling pathway to downregulate the expression of *CYP11A1* and *CYP19*, decrease placental aromatase activity, and cause the estradiol and progesterone production to decrease [47]. A lack of hormones may contribute to placental insufficiency and pregnancy failure. BPA also inhibits the expression of Wnt2/β-catenin and increased DNA methylation of Wnt2 in its promoter region, thereby mediating epigenetic modification [48]. These findings suggest a potential mechanism by which BPA causes reproductive toxicity. The possible mechanisms of BPA in the placenta are complicated and worth exploring.

Adverse effects of low doses of BPA exposure during pregnancy on the female reproductive system have been reported [49]. In addition, BPA may enter the placenta through the mother, so mammals’ prenatal exposure to low concentrations of BPA can adversely affect the developing fetus. When placental cells were exposed to seven different concentrations of BPA for one day, researchers discovered that damage to the cell membrane was 1.3 to 1.7 times higher in placenta cells than untreated cells. Such exposure can lead to adverse pregnancy outcomes such as premature birth, preeclampsia, and pregnancy loss [50]. A study demonstrated for the first time that exposure of BeWo cells to BPA at a concentration of 50 nM increased the gene expression of three human endogenous retrovirus capsules and related syncytia proteins, thereby affecting the fusion of trophoblast cells and endocrine activity [51]. As observed in mice, over-promotion of syncytial trophoblast may be detrimental to placental mammals, unbalancing the trophoblast lineage during development, and then affecting fetal growth. Furthermore, BPA can significantly induce the secretion of β-hCG, enhancing the endocrine function of the placenta, and inhibiting trophoblast invasion [52]. In addition, some findings demonstrated that exposure to BPA can alter the expression of the imprinted genes, leading to abnormal placental development, and also reduce DNA methylation in the mouse placenta to perturb fetal health via epigenetic changes [53]. Maternal exposure to BPA in early pregnancy predicts DNA methylation in newborns, which is associated with gene expression levels related to growth and metabolism. And DNA methylation has a potential impact on disease susceptibility in the fetus [54]. Female mice exposure to BPA during early pregnancy can result in impaired remodeling of uterine SAs, which has a negative effect on vasoconstrictive ability and blood flow velocity, limiting the blood supply to the placenta and fetus. The insufficient remodeling of uterine SAs caused by BPA exposure not only reduces the weight of placenta but also affects fetal growth and provokes intrauterine growth retardation (IUGR) in the fetus [55].

Newborns are susceptible to BPA because of an immature liver function and underdeveloped glucose acidification movement. BPA acts as an androgen receptor (AR) antagonist to inhibit dihydrotestosterone (DHT) induced transcriptional activity, thereby affecting the development and function of the male reproductive systems. Pregnant mice exposed to 10 μg/kg BPA per day will lead to abnormal development of the prostate and urethra in male fetuses and permanently disrupt cellular control systems and increase the risk of prostate cancer in adult male mammals [56,57]. Subsequently, in adulthood, the number of prostatic androgen receptors per cell doubled, causing an enlarged prostate [58]. The developing brain is particularly sensitive to BPA; rats’ exposure to a dosage less than 50 μg/kg BPA of the human acceptable daily intake level can affect the sexual differentiation of the neural structures and behavior of the fetus [59]. BPA alters intracellular molecular mechanisms that may cause breast cancer by binding to estrogen-related receptors (ERRs) or G protein-coupled receptors (GPERs). Fetal exposure to low doses of BPA has long-term effects, increasing the risk of breast cancer in adulthood [60].

### 3.2. Organochlorine Pesticides 

Organochlorine pesticides (OCPs) are a class of organic compounds containing carbon, hydrogen, and chlorine, which are used to control insects, weeds, and fungus. Statistically, in the use of different pesticides, 40% of all pesticides used belong to the organochlorine class of chemicals. This is a large group of chlorinated hydrocarbons that includes dichlorodiphenyltrichloroethane (DDT) and its metabolites, hexachlorobenzene HCB, lindane, and dieldrin [61]. DDT is widely used in agriculture but is now forbidden in many countries. OCPs have the features of low water solubility, stable chemical properties, and an ability to adsorb organic materials in soil; thus, the use of a large number of pesticides in agricultural production has led to the aggravation of soil, water, and air pollution. OCPs are gradually transmitted to fish and higher animals such as humans through the food chain, therefore, many aquatic and terrestrial species are seriously affected [62,63].

Organic chloride pollutants have a high affinity with hydrocarbon (Ah) receptors [64]. The combination intensely induced cytochrome *P4501A1 (CYP1A1)* gene encoding the cytochrome P-450 1A1 enzymes involves organochlorines’ metabolism [65]. Organochlorine metabolism in the body can produce a large number of reactive oxygen species, which the anti-oxidant system fails to eliminate, thereby breaking DNA strands and affecting mitochondrial function. CYP1A1 is involved in metabolism in the human placenta; abnormal expression of this enzyme may disrupt the placental detoxification machinery [66,67].

It is of concern that organochlorine pesticides often badly affect non-target species, such as human beings and animals, when applied to their target organisms. Organochlorine pesticides have a lipophilic nature that can easily dissolve in human and animal adipose tissue. During pregnancy, they can be mobilized and enter maternal blood circulation and reach the placenta [68]. These compounds can interfere with the functions of the placenta, such as the production and release of hormones and enzymes, the transportation of nutrients, and the generation of waste, and then disrupt fetal development and the last stage of placental life [69]. A result of research showed that oxidative stress caused by organochlorine pesticides can result in preterm deliveries [70]. Pathak et al. have observed that the higher concentration of β-HCH in umbilical cord blood increased the risk of premature birth. Furthermore, they also noticed that the serum level of γ-HCH was positively correlated with female habitual miscarriage [71,72]. Exposure to organic pollutants during pregnancy increased p,p’-DDE concentrations in maternal blood, and the levels of p,p’-DDE transferred from maternal blood to breast milk and umbilical cord blood was also increased. It may have toxic effects on breast and placental functions, such as vascular lesions and endothelial degeneration [73]. In the epidemiological model, the levels of maternal organochlorine pesticides were associated with hyper-methylation of placental genes, and high levels of p,p’ -DDT are significantly associated with insulin-like growth factor 2 (IGF2), which may also explain fetal growth restriction [74]. Dewan et al. found OCP levels in placentas were negatively correlated with chest circumference, head circumference, and body mass index in newborns [75].

It was reported that early exposure to organochlorine compounds with endocrine-disruption activity may interfere with neonatal thyroid hormone status [76]. OCPs in maternal blood can be transported through the placenta and affect neonatal thyroid hormone levels [77]. By investigating the thyroid hormone situation of 15-year-old children in rural areas of Brazil with severe OPC exposure, it was found that the increase of total triiodothyronine (T3) levels in serum was related to the high concentration of OCPs, which had adverse impacts on children’s growth [78]. Moreover, maternal exposure to OCPs in early pregnancy contributed adversely to sperm density and activity in male fetuses, resulting in a decline in male fertility [79]. OCPs act as endocrine-disruptor chemicals by disrupting the physiologic function of endogenous hormones and thus possibly increasing cancer risk. Kumar et al. revealed a significant positive correlation between the risk of prostate cancer and serum levels of OCPs, especially the high level of β-HCH, γ-HCH, and p,p’-DDE [80].

### 3.3. Diethylstilbestrol

Diethylstilbestrol, a synthetic non-steroidal estrogen substance, produces the pharmacological and therapeutic effects of natural estradiol but is five times more potent [81]. DES can promote the normal development of the female reproductive system, endometrial hyperplasia, and vaginal epithelial keratinization and improve the sensitivity of the uterus to oxytocin. When low doses of DES are used, it stimulates the secretion of anterior pituitary gonadotropins and prolactin. Conversely, the use of high doses of DES will inhibit this effect. In addition, DES also has an anti-androgenic effect and binds to estrogen receptors (ERs) or progesterone receptors (PRs), which has adverse effects on reproduction. Therefore, in the 1970s, DES was widely used as an estrogen-like drug for oral contraceptives, preventing miscarriage, and replacing hormones for clinical treatment. However, in 1971, research found for the first time that vaginal malignancies in adolescent girls were partially attributed to DES exposure during fetal development [82]. Subsequent studies have shown that the approval of DES for clinical treatment was a serious mistake, which has resulted in a series of irreparable consequences [83]. 

Several ongoing studies have compared the health status of women exposed to DES and unexposed controls of the corresponding age. These studies found that the incidences of infertility, spontaneous miscarriage, and premature delivery, pregnancy loss in the second trimester, ectopic pregnancy, pre-eclampsia, and stillbirth in women with DES exposure were much higher than those in the control group. It is worth noting that the risk of breast cancer in women experiencing DES exposure is also increasing [84,85,86,87]. As we have mentioned before, exposure to EDCs may lead to enormous and unrecoverable damage to the fetus. Daughters of DES-treated women were affected by DES in the uterus, which may cause vaginal adenoma, corpus luteum deficiency, polycystic ovary induction, and other reproductive abnormalities [88,89,90]. Sons of DES-treated women has a higher rate of genital tract abnormalities [91].

DES affects mothers and their daughters/sons, not only at a particular physiological stage, but also with a long-term impact [2]. Studies have shown that the presence of DES has affected multiple signaling pathways in the body, but this is not yet fully clarified. It is now commonly believed that early DES exposure causes its effects by genetic and epigenetic alterations, not by changes in gene mutation [92]. Neonatal exposure to DES can cause extensive changes in gene expression that persist into adulthood. Through molecular studies, many of the changes caused by DES is the control of reproductive tract development by inhibiting HOX and Wnt genes, and the DES induced uterine malformations may be due to the downregulation of HOXA11 gene [93,94]. Due to exposure to DES may cause DNA methylation, the fetal genome is allowed to encode multiple defects, which may not be expressed before a certain period of time (puberty, for example) [95]. Studies have shown that microRNA levels may change due to the effects of DES, and microRNA-mediated upregulation of Fas and its ligands leads to increased autophagy and apoptosis [96]. 

### 3.4. Phthalates

Phthalates (PAEs) are industrial chemicals that are widely used as plasticizers to increase the flexibility and softness of plastic and are also used as stabilizing agents. A wide variety of consumer products are made with phthalates, including personal care products, automotive parts, children’s toys, food packaging, and building materials [97]. In addition, some medical equipment also contains phthalates. As an endocrine disruptor, phthalates have been listed as a major environmental estrogen with a function similar to artificial hormones and are the most widespread pollutant. Studies of phthalates both in vivo and in vitro have shown that they have a higher level of biological activity and toxicity when they are metabolized as monoesters [98].

**Figure 3:**
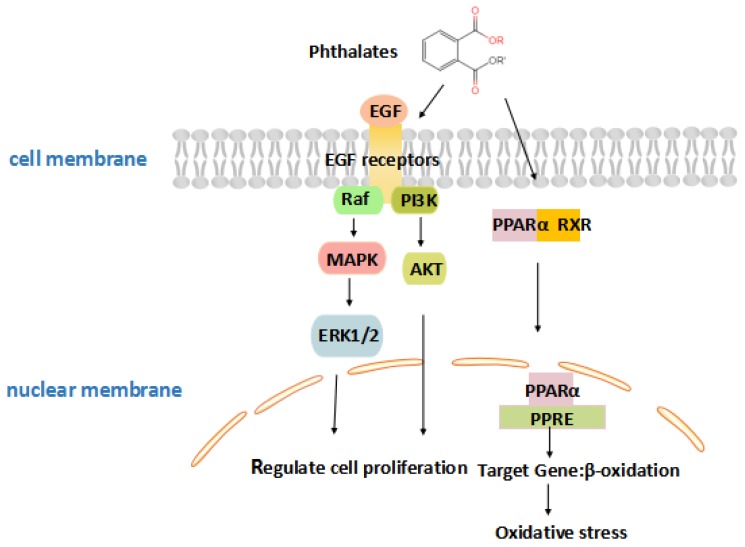
A schematic illustrating the effects of phthalates on placental growth. Phthalates activate peroxisome proliferator-activated receptor (PPAR), which is associated with placental development, or modulate the combination of epidermal growth factor (EGF) and epidermal growth factor receptor (EGFR), and activate phosphatidylinositol 3-kinase/protein kinase B (PI3K/Akt) and signal-regulated kinase (ERK) signaling pathways, then regulate proliferation, survival, and differentiation in the body.

Phthalates are important synthetic chemicals which can induce toxicity, and the mechanism of the toxicity caused by phthalates needs to be further understood. Phthalates increase reactive oxygen species to alter the expression and activity of antioxidant enzymes that result in DNA damage [99]. As detailed in Figure 3, monoethylhexyl phthalate (MEHP), the reactive metabolite of phthalates, activates peroxisome proliferator-activated receptor (PPAR), and then combines with retinoid X receptor (RXR) [100]. Subsequently, they are transported to the nucleus, inducing gene expression and increasing oxidative stress. Oxidative DNA damage could inhibit the binding of methyl-CpG binding proteins (MeCP) and lead to epigenetic changes in chromatin organization [101]. MEHP exposure, inhibiting trophoblast invasion by activating PPARγ, is the most critical mechanism for early pregnancy loss [102]. Furthermore, placental ErbB signaling is one of the most important signaling pathways in response to DNA methylation and gene expression induced by phthalate exposure. This pathway works through tyrosine kinases, including two critical members, epidermal growth factor (EGF), and epidermal growth factor receptor (EGFR). The EGF signaling pathway activates numerous intercellular pathways that stimulate placental growth and function, including regulation of trophoblast cell proliferation, differentiation, and invasion [103,104]. Exposure to phthalates in early pregnancy alters placental transcriptome as a methylation change of critical placental DNA such as Insulin Growth Factor 2 (IGF2) [105]. This suggests that the pathway may be a potential target for endocrine disruption by phthalates.

Owing to the wide application of PAEs, problems such as preterm birth, ovarian cancer, and recurrent pregnancy loss are becoming increasingly prominent [106,107]. A previous study examined urine samples of human chorionic gonadotropin to investigate a possible link between phthalate exposure and early miscarriage. Results showed that exposure to high doses of MEHP was associated with early miscarriage compared to low MEHP exposure [108]. Jukic et al. revealed that pregnant females’ exposure to excess di(2-ethylhexyl) phthalate (DEHP) was believed to increase the risk of developing early embryo loss [109]. MEHP changed the synthesis of lipidosomes in the JEG-3 human trophoblast cells leading to lipid imbalance [110]. Furthermore, Grindler et al. investigated the methylation changes of 39 genes in first trimester placenta and found that most of the changes were negative [105].

Phthalates can cross the placental barrier as a result of continuous exposure during pregnancy, with profoundly negative consequences for the future health of infants, by increasing the risk of developing defects such as low birth weight, cardiovascular diseases, cryptorchidism, and cancer later in life [111,112]. The metabolites of DEHP have been detected by mass spectrometry in amniotic fluid. The results showed that the metabolism may be associated with cryptorchidism and hypospadias in male fetuses [113].

### 3.5. Phytoestrogens

Phytoestrogens are compounds that have weak estrogenic effects in plants and are known to be present in several different types of food, such as grains, beans, fruits, vegetables, and, in particular, many soy products [114,115]. The three major classes of phytoestrogens in the human body are lignan, isoflavones (daidzein, genistein, and glycitein), and coumestans (coumestrol). Phytoestrogens are a plant component with biological activity similar to animal estrogen, with a wide range of effects on hormone-related diseases, and they exert a weak estrogen-like effect by binding to the ER with low affinity. ERs are present in different tissues, including the reproductive tract, bones, placenta, and mammary gland, suggesting that phytoestrogens may play a tissue-specific hormonal role [116,117]. ERα is perceived to promote cell growth, whereas ERβ does the opposite, mainly promoting apoptosis [118]. Different phytoestrogens have different affinities for binding to ERs. Coumestrol has the highest affinity, which is 10 to 20 times lower than estradiol, followed by genistein. The same phytoestrogens have a different affinity for ERα and ERβ. The affinity of apigenin for ERβ is higher than that of ERα. The affinity of coumestrol and genistein for ERβ is higher than for ERα.

For example, genistein is a major active factor and the most effective functional component of soy isoflavones, with a variety of physiological functions. The genistein can alter the level of IGF-1 and placenta growth factor (PGF), and thus alter the normal growth and development of the placenta and the fetus [119]. Genistein and daidzein could inhibit the production of hCG, and the production of hCG was significantly decreased in the trophoblast cells treated with genistein and daidzein [120]. Genistein and daidzein are type of phytoestrogens, so exposure to these estrogen-like compounds during sensitive periods of growth and development may have the capacity to alter the function of the reproductive system, thereby affecting fetal health.

## 4. Concluding Remarks

This review details the characteristics, mechanisms, validation methods, and influences on the placenta and the fetus of several kinds of endocrine disrupting chemicals, including bisphenol A, organochlorine pesticides, diethylstilbestrol, and phthalates. Today, there are numerous ways for people to directly or indirectly come in contact with EDCs. The negative impact of the toxicity of EDCs on human health is a serious problem that must be addressed. Some EDCs can cross the placental barrier and accumulate in the placenta. As the most important and significant development stage of life, the fetus should be protected from all toxic and harmful substances. Further research on EDCs is ongoing, but the unknown mechanisms of EDCs’ functions remain a challenge for scientists to discover and understand. The effects of EDCs on the early placenta may raise concerns about the proper use of products containing EDCs during pregnancy and encourage early preventative measures. We hope this brief review provides a reference for human health and disease.

## Figures and Tables

**Table 1:** Detection methods of some endocrine disruptors in maternal body fluids.

EDCs Exposure	Compound	Area	Measurement Method	Body Fluid	Mean ± SD or Range	Reference
Bisphenol A	BPA	Germany	Chemical derivatization-GC-MS	Maternal plasma	0.3–18.9 ng/mL	[31]
	BPA	Germany	GC-MS	Fetal plasma	0.2–9.2 ng/mL	[31]
	BPA	Japan	Enzyme-linked immunosorbent assay	Amniotic fluids	0–8.38 ng/mL	[32,33]
	BPA	Germany	GC-MS	The placental tissue	1–104.4 ng/g	[31,34]
Organochlorine pesticides	α-hexachlorocyclohexane (α-HCH)	The Los Angeles area	GC-MS	Amniotic fluids	0.15 ± 0.06 ng/mL	[35]
	p,p′-Dichlorodiphenyldichloroethylene (p,p’-DDE)	The Los Angeles area	GC-MS	Amniotic fluids	0.21 ± 0.18 ng/mL	[35]
	p,p’-Dichloro-diphenyl-trichloroethane (p,p′-DDT)	Mexico	Dual column gas chromatograph-electron capture detector	Serum	676 ng/g-lipid	[36]
	p,p′-DDE	Mexico	Dual column gas chromatograph-electron capture detector	Serum	4,843 ng/g-lipid	[36]
Phthalates	Methylerythritol cyclodiphosphate (MECPP)	America	Isotope-dilution HPLC/MS/MS	Breast milk	0.1–0.4 μg/L	[37]
	MEHHP	America	HPLC/MS/MS	Breast milk	0.2–0.3 μg/L	[37]
	Mono-ethyl phthalate (MEP)	Canada	LC-MS/MS	Urine	32.02 μg/L	[38]
	Mono-n-butyl phthalate (MnBP)	Canada	LC-MS / MS	Urine	11.59 μg/L	[38]
Diethylstilbestrol	DES	China	HPLC with diode array detection	Urine	1–200 μg/L	[39]
Phytoestrogens	Daidzein	America	GC/MS	Amniotic fluid	1.44–5.52 ng/mL	[40]
	Genistein	America	GC/MS	Amniotic fluid	1.69–6.54 ng/mL	[40]
	Daidzein	America	Ultra-performance liquid chromatography-MS/MS (UPLC-MS/MS)	Urine	0.11 μg/L	[41]
	Genistein	America	UPLC-MS/MS	Urine	0.59 μg/L	[41]
